# Pattern of Gustatory Impairment and its Recovery after Head and Neck Irradiation

**Published:** 2017-11

**Authors:** Preety Negi, Pamela-Alice Kingsley, Maria Thomas, Jaineet Sachdeva, Himanshu Srivastava, Babusha Kalra

**Affiliations:** 1 *Department of Radiation Oncology, Christian Medical College & Hospital, Ludhiana, Punjab, India.*; 2 *Department of Biochemistry, Christian Medical College & Hospital, Ludhiana, Punjab, India.*; 3 *Department of Radiation Oncology, Capitol Hospital, Jalandhar, Punjab, India. *

**Keywords:** Chemoradiotherapy, Dysgeusia, Head and neck neoplasms, Quality of life, Taste threshold

## Abstract

**Introduction:**

The majority of patients receiving concurrent chemoradiotherapy frequently complain of changes in their taste perception, and other distressing symptoms affecting their quality of life. This study was undertaken to determine the pattern of gustatory impairment and its recovery in irradiated head and neck cancer patients in India.

**Materials and Methods:**

Thirty patients undergoing radical head and neck irradiation were enrolled and assessed for the four basic taste quality (sweet, salt, sour and bitter) by a forced three-choice stimulus drop technique measuring their taste recognition thresholds at baseline, weekly during radiation therapy (RT) and every month for 6 months following completion of RT.

**Results:**

The maximum taste loss for any taste quality developed after the third week of RT. Irrespective of the taste quality, the majority of patients developed their maximum taste loss in the fourth to sixth week. The maximum taste loss was highest (100%) for the bitter taste and least (40.7%) for the sweet taste. Taste recovery for sweet, salt and sour taste qualities started from the first month onwards, but not for bitter taste. All taste qualities were severely affected in patients with primary involvement of the oral cavity and oropharynx as compared with nasopharynx, hypopharynx and laryngeal tumors.

**Conclusions:**

Taste dysfunction is a frequently ignored adverse effect of head and neck cancer treatment, seriously affecting the patient’s quality of life. Clinicians must make patients aware of this specific gustatory dysfunction and its pattern of recovery. Future efforts should be directed towards minimizing this dysfunction, specifically in tumors arising from the oral cavity and oropharynx.

## Introduction

Concurrent chemoradiation therapy has emerged as the definitive treatment, as well as an adjuvant treatment option, for patients with locally advanced squamous cell carcinoma of the head and neck (1). Ionizing radiation is known to cause damage to the normal tissues located within the radiation portals, and the intensity of this damage differs in individual patients. Gustatory dysfunction has been recognized as a persistent problem across various forms of head and neck cancer treatments, sites, and stages (2). Taste alteration is considered a distressing symptom leading to malnutrition and resulting in significant morbidity affecting the daily quality of life in a large proportion of patients. Alteration in taste sensation arises within days of initiation of radiation therapy (RT), and becomes increasingly problematic beyond doses of 20 Gy. Approximately 90% of patients who have received 60 Gy are likely to have significant gustatory dysfunction (3). This loss of gustatory function is generally temporary, with a gradual recovery to normal levels within a year of RT; although in some patients this recovery period may extend up to 5 years after RT (4). Gustatory dysfunction is often ignored by both the clinician and the patient, compared with other adverse effects of radiation treatment. Few prospective studies have been performed investigating the four basic taste qualities (sweet, salt, sour and bitter) in head and neck cancer patients receiving RT. This study was designed to determine the pattern of gustatory impairment and its recovery in patients receiving head and neck cancer RT.

## Materials and Methods

This prospective study included 30 cases of squamous cell carcinoma in the head and neck region (with primary sites involving the oral cavity, oropharynx, nasopharynx, hypopharynx, or larynx) from a period extending from December 2013 to November 2014. All patients who were intended for head and neck RT were screened for participation in this study. Eligible patients were those with an Eastern Cooperative Oncology Group Performance Status ≤3, without any coexisting or prior malignancy in the head and neck region, and those not suffering from any intercurrent illnesses affecting the salivary function (such as Sjogren’s syndrome or human immunodeficiency virus). Patients who had undergone total or partial glossectomies or who were on taste-altering medications (biologic therapies such as interleukin-2 and interferons, anti-rheumatic drugs, penicillamine, angiotensin-converting enzyme [ACE] inhibitors or clarithromycin) were excluded. Patients fulfilling the study participation criteria were enrolled in the study after signing an informed consent form.

As per our institutional guidelines, the study protocol was approved by a research committee and institutional ethics committee. All patients underwent a thorough clinical examination and were subjected to the following investigations: complete blood count, renal function tests, direct laryngoscopy and biopsy, contrast-enhanced computed tomography (CT) scan of head and neck, chest X-ray, and ultrasound abdomen.

A spiral planning CT scan with contrast was obtained from the base of the skull to the second intercostal space using a 128-slice Philips ingenuity High Speed CT machine (slice thickness, 3 mm) with immobilization using a custom-made head-to-neck thermoplastic cast and neck support. All patients received three-dimensional conformal RT with a 6 MV linear accelerator. Target volumes were delineated slice-by-slice on treatment planning scans using Radiation Therapy Oncology Group guidelines. Plans were generated using the treatment planning system, CMS XiO. Prescribed doses were 60–70 Gy at 2 Gy / fraction once daily, 5 days a week with concurrent chemotherapy with weekly cisplatin 40 mg/m^2^ or 3-weekly cisplatin 100 mg/m^2^.

*Taste function tests:*

Taste function was determined for all four basic taste qualities. The taste function test was based on the standard forced three-choice stimulus drop technique (5) for the four basic taste qualities. Sodium chloride was used for the salt taste, purified sucrose for sweet taste, hydrochloride for sour taste, and urea was used for the bitter taste. Each basic taste test solution was prepared with distilled water.

*Forced three-choice stimulus drop technique:*

This technique consisted of introducing sequences of three drops of solutions into the oral cavity. Two of the drops were water, and one was a solute dissolved in water. The solutes used were sucrose (60 mM/L to 1000 mM/L), NaCl (60 mM/L to 3000 mM/L), HCl (6 mM/L to 500 mM/L) and urea (150 mM/L to 8000 mM/L) (Table.1).

**Table 1:** Different concentrations of solutes used

**Solute**	**Concentrations used**					
Sucrose	60	90	150	500	800	1000
NaCl HCl	60 6	90 30	150 60	500 150	1000 300	3000 500
Urea	150	300	800	1000	5000	8000

Gradually different concentrations were tested in each patient in ascending order until they were able to sense the taste. Patients were told to rinse their mouth with water in between the different solutions. Each solution was tasted only once. After tasting the solution, the patient responded whether the solution tasted neutral (as water) or had one of the four basic taste qualities; i.e. sweet, salt, sour or bitter. The lowest concentration of solute that the patient consistently recognized correctly as sweet, salt, sour or bitter was called the recognition threshold. 

These taste recognition threshold measurements were performed once before treatment, weekly during treatment, and every month up to 6 months after completion of treatment.

Scoring system for taste loss (for all taste qualities) (5):

Score 0 – Total taste loss for any given taste quality (Total taste loss).

Score 1– Detect and recognize a taste quality at the strongest concentration of salute used only (Serious taste loss).

Score 2 – Detected and recognized the taste quality at the middle concentration of solute (Moderate taste loss).

Score 3-Detect and recognize the taste quality at all concentrations (No taste loss).

Maximum taste loss included patients with total and serious taste loss.

*Statistical Analysis:*

Categorical variables are presented as numbers and percentages (%). Qualitative variables were correlated using the Chi-Square test / Fisher’s exact test. 

A *p*-value of <0.05 was considered statistically significant. The data were entered in an MS Excel spreadsheet and analysis was performed using the Statistical Package for Social Sciences (SPSS) version 21.0.

## Results

A total of 30 eligible patients were enrolled in this study. Of these, three patients did not complete the study, and hence only 27 patient data were included in the final analysis. One patient died from non-oncologic disease and two were lost to follow-up. The mean age of the patients was 55 ± 14.11 years (Table.2).

**Table 2:** Clinical and demographic details

**Patient characteristics **	**No. of patients (n=27)**
Age	
Mean age	55 ±14.11 years (range: 32–80)
Gender	
Male	22 (81.5%)
Female	5 (18.5%)
Primary tumor	
Oral cavity	8 (29.6%)
Oropharynx	6 (22.2%)
Nasopharynx	2 (7.4%)
Hypopharynx	5 (18.5%)
Larynx	6 (22.2%)
T-Stage	
T1	2 (7.4%)
T2	7 (25.9%)
T3	10 (37%)
T4	8 (29.6%)
N-Stage	
N0	10 (37%)
N1	5 (18.5%)
N2	10 (37%)
N3	2 (7.4%)

None of the patients had total taste loss prior to initiation of RT. However, prior to RT, 29.6%, 33.3%, 24.1% and 22.7% patients had a taste loss for bitter, sweet, salt and sour taste qualities, respectively.

Before the third week of RT, none of the patients had maximum taste loss for any taste quality. Maximum taste loss was observed in the fourth to the sixth week of RT, irrespective of the taste quality. Twenty-seven (100%) patients had a maximum taste loss for the bitter taste during the seventh week of treatment. This was followed by the salt (77.8%) and sour tastes (70.4%). Only 40.7% patients had maximum taste loss for the sweet taste quality during the treatment period (Fig. 1). Total taste loss was most pronounced for the bitter taste (55.6%) and least pronounced for the sweet taste (37%) (Fig. 2).

Recovery of taste for sweet, salt and sour taste qualities started from the first month onwards, except for the bitter taste. The sweet taste showed the maximum taste loss at the fourth week of RT (P=0.0005). The quickest recovery was observed for the sweet taste, with the maximum number of patients recovering by the third month after RT, except one (P=0.001). The salt taste was the most affected at the fifth week of RT (P=0.0001), and the majority of patients recovered by the fourth month (P=0.0002). Recovery of the sour taste occurred after the sixth week of RT (P=0.01), and most patients recovered by the fourth month (P=0.01). Among all the taste qualities, maximum taste loss was most pronounced at the fourth week of RT (P= 0.14). Recovery of the bitter taste did not occur in 25.9% of patients (P<0.0001), and 18.5% and 11.10% of patients did not recover the salt and sour taste, respectively (Fig. 3).

To explore any relationship between the primary site of origin and gustatory dysfunction, we substratified the patients in Group A and Group B. Group A consisted of patients with cancers of the oral cavity and oropharynx and Group B included patients of cancers of the nasopharynx, hypopharynx and larynx. On comparing all the taste qualities for both the groups, we found that all taste qualities were severely affected in Group A patients compared with Group B patients. This difference, according to the primary tumor site, almost reached statistically significant level for the sweet (P= 0.05) and salt (P= 0.05) taste qualities (Fig.4). Patients were divided according to age group as <50 years and ≥50 years. Both taste loss and its recovery were independent of age grouping for all four taste qualities (P= NS).

## Discussion

The four primary taste qualities described in the literature are sweet, salt, sour and bitter, as mediated by the taste buds. The gustatory epithelia are located in the edges and dorsal surface of the tongue, soft palate, and pharynx (6). Knowledge of radiotherapy-induced gustatory dysfunction is of utmost importance since it is a common side effect of RT. Unfortunately, it is frequently an overlooked condition, receiving relatively less medical and clinical research attention (7). Our study addresses an important issue of awareness towards gustatory dysfunction after head and neck irradiation, which is still not a priority in our daily clinical practice.

We found that a certain percentage of patients had gustatory dysfunction, even prior to treatment. One review suggested that up to 89% of patients prior to RT have some gustatory dysfunction (8). Common gustatory tests have shown some evidence for decreased taste sensitivity in cancer patients, and these alterations may continue even after the completion of cancer treatment (9). RT induces cell cycle arrest and apoptosis of the taste progenitor cells. This cessation of proliferation is responsible for the reduced influx of new cells into the taste buds following irradiation, thereby damaging the taste buds (10). The detection of taste is influenced by the diffusion of taste substances to the taste receptors, chemical interaction with taste substances and changes in the sensitivity of the taste receptors (11). Other predisposing factors for gustatory impairment during RT include oral mucositis and salivary hypofunction (12).

Altered gustatory function is a transient phenomenon during and after RT. Most studies have recognized bitter as the basic taste most influenced by cancer and its treatment (13–15). When our results were compared to those in the literature, we noticed that all (100%) patients had taste loss for the bitter taste, while sweet (40.7%) was the least affected taste in terms of maximal taste loss. This finding is similar to that observed in other studies that also reported bitter and salt qualities being affected the earliest and to the greatest extent (3,16,17).

The incidence of gustatory dysfunction during the course of curative head and neck RT becomes measurably impaired during the first week of treatment, worse during the second week, and usually peaks around third to the fifth week of irradiation (19–21). More than 90% of patients lose their taste sensitivity after receiving 6000 cGy of irradiation for head and neck cancer (22). We found that maximal taste loss became measurably impaired after the third week of RT for all the taste qualities. Maximum taste loss was observed to occur in the fourth to the sixth week, irrespective of the taste quality.

Earlier researchers considered that certain areas on the tongue are more sensitive than others to specific tastes (21,23), although concerns have been expressed about this notion in view of the lack of scientific basis. Further studies have clarified that taste is mediated by specialized epithelial cells distributed throughout the oral cavity, palate, lips, cheeks, pharynx, epiglottis, larynx, and the upper part of the esophagus (18,24). On categorizing the patients according to the primary tumor site, we found that all taste qualities were severely affected in patients with a cancer primary involving the oral cavity and oropharynx as compared with those with cancer of the nasopharynx, hypopharynx or larynx. A plausible explanation would be damage to the normal tissues located in the radiation portals, specifically the volume of the tongue in the irradiated field. This finding is consistent with a previous study by Sapir et al. (25) in 73 patients who reported dysgeusia after RT to the oral cavity. The authors observed that taste impairment significantly correlated with mean radiation dose to the oral cavity, and proposed that reduction in oral cavity dose is likely to improve dysgeusia. Additionally, in a prospective study conducted at the Royal Marsden Hospital and the Queen Elizabeth Hospital in 26 head and neck carcinoma patients undergoing radical irradiation, the volume of tongue contained within the high-dose volume of the radiation treatment field correlated with the degree of taste loss. The results of their study suggested a significant association between the proportion of tongue within the treatment field and subjective as well as objective taste loss (26).

Recovery of gustatory dysfunction following treatment is variable. Based on the results of several studies, improvement in gustatory function can occur within 2–6 months, or may continue indefinitely, for as long as up to 7 years (3,8,17,27,28). In the present study, the quickest recovery was noticed for the sweet taste, with the maximum number of patients recovering within the first 3 months, while the bitter taste had the minimal recovery in the specified follow-up period. None of the taste qualities recovered completely within 6 months. In contrast to this, a study in 13 patients with a primary tumor of the oropharynx reported significant elevations in thresholds for sweet (P<0.005), salt (P< 0.005), bitter (P<0.005) and sour (P<0.001) during RT, which were restored to baseline levels at 6 months and 1 year after RT. This study demonstrated that recovery of radiation-induced taste deficits can occur by 6 months (17).

RT-induced gustatory dysfunction is a common symptom, and is under reported in the majority of the patients. Limited data have been published so far in this regard in Indian patients. Several studies have revealed that gustatory dysfunction contributes to poor dietary intake, leading to subsequent weight loss, potentially negatively affecting the treatment outcome and patient quality of life (8,29,30). A modified diet and pre-counseling of gustatory dysfunction helps in achieving a better quality of life. This study is probably one of the few studies in which the effect of RT on gustatory function has been tested in head and neck cancer patients. These findings offer a starting point to help in educating patients regarding gustatory dysfunction and its recovery pattern, since a well-informed patient can deal better with the treatment sequelae.

## Conclusions

This study provides much-needed insight into a common, yet not extensively studied, complication of RT-induced gustatory dysfunction in head and neck cancer patients. It is of paramount importance that, as treating oncologists, we actively educate the patient regarding gustatory dysfunction, thereby helping patients navigate rigorous treatment with a quick recovery. We observed the maximum taste loss for the bitter taste, followed by the salt and sour taste, while the sweet taste was least affected. Maximum taste loss was noticed during the fourth to sixth week of RT, irrespective of the taste quality. Quickest recovery was observed for the sweet taste, while the bitter taste showed the least recovery. Further multi-institutional research with larger samples is needed for a better understanding of gustatory dysfunction in an Indian subset of patients, thereby validating these results.
